# Small diameter vascular grafts: progress on electrospinning matrix/stem cell blending approach

**DOI:** 10.3389/fbioe.2024.1385032

**Published:** 2024-05-14

**Authors:** Nuoxin Wang, Jiajing Chen, Qingqing Hu, Yunfeng He, Pu Shen, Dingkun Yang, Haoyuan Wang, Dong Weng, Zhixu He

**Affiliations:** ^1^ Key Laboratory of Cell Engineering of Guizhou Province, Affiliated Hospital of Zunyi Medical University, Zunyi, China; ^2^ The Clinical Stem Cell Research Institute, Affiliated Hospital of Zunyi Medical University, Zunyi, China; ^3^ Collaborative Innovation Center of Chinese Ministry of Education, Zunyi Medical University, Zunyi, China; ^4^ The First Clinical Institute, Zunyi Medical University, Zunyi, China; ^5^ Department of Cardiothoracic Surgery, The Second Affiliated Hospital of Zunyi Medical University, Zunyi, China; ^6^ The Second Clinical Institute, Zunyi Medical University, Zunyi, China; ^7^ Department of Pediatrics, Affiliated Hospital of Zunyi Medical University, Zunyi, China

**Keywords:** small-diameter vascular graft, electrospinning, stem cell, blend material, regeneration

## Abstract

The exploration of the next-generation small diameter vascular grafts (SDVGs) will never stop until they possess high biocompatibility and patency comparable to autologous native blood vessels. Integrating biocompatible electrospinning (ES) matrices with highly bioactive stem cells (SCs) provides a rational and promising solution. ES is a simple, fast, flexible and universal technology to prepare extracellular matrix-like fibrous scaffolds in large scale, while SCs are valuable, multifunctional and favorable seed cells with special characteristics for the emerging field of cell therapy and regenerative medicine. Both ES matrices and SCs are advanced resources with medical application prospects, and the combination may share their advantages to drive the overcoming of the long-lasting hurdles in SDVG field. In this review, the advances on SDVGs based on ES matrices and SCs (including pluripotent SCs, multipotent SCs, and unipotent SCs) are sorted out, and current challenges and future prospects are discussed.

## 1 Introduction

### 1.1 Small-diameter vascular grafts: a demanding clinical need

In recent years, due to the continuous acceleration of population aging and urbanization, the people suffering cardiovascular diseases (CVDs) worldwide boost rapidly, which have become the primary cause of death all over the world (Shi et al., 2022). Among CVDs, end-stage severe obstructive ones such as coronary heart disease and severe lower limb ischemia, are very risky, but their preferred treatment, the vascular transplantation, is largely restricted by lacking enough available small diameter vascular grafts (SDVGs, inner diameter≤6 mm) (Wei et al., 2022). At present, autologous blood vessels (the most popularly used ones include great saphenous veins, internal mammary arteries, and radial arteries) are considered the gold standard graft for vascular transplantation due to their high biocompatibility with patients and high long-term patency rate after transplantation. However, invasive acquisition of these autologous vessels would lead to significant damage and complications for the patients and more importantly, due to the patients’ health conditions and/or vascular damages, around one-third of the patients’ autologous vessels are difficult to obtain (Seifu et al., 2013). Considering the large population of patients over the world, the quantity gap for SDVGs is a critical problem, and therefore it is urgent to develop SDVG suitable for clinical transplantation. At present, large-diameter vascular grafts (inner diameter>6 mm) based on synthetic materials such as expanded polytetrafluoroethylene, polyester, and natural silk have been successfully applied in clinical practice (e.g., in aortic replacement). However, SDVGs prepared by the aforementioned materials face the challenge of acute thrombosis, lumen narrowing, and intimal hyperplasia (IH) after transplantation, because of their narrow caliber, slow flow rate, and complex local blood flow environment. Hence, clinically available SDVGs have not been achieved.

### 1.2 Electrospinning: a favorable preparation method for SDVGs

The existing preparation methods for SDVGs mainly include: decellularization, dipping/leaching, coagulation, layer-by-layer self-assembly, three-dimensional (3D) printing, electrospinning (ES), and so forth. The basic principle of the decellularization method is using physical methods or ionic solvents to efficiently rid out of the cells from the extracellular matrix (ECM), while retaining the main components of the ECM with biological activities as much as possible. However, the process is rather complex, which would inevitably destroy the integrity of the ECM and leave certain immunogenic substances, thus affecting the overall performance of the scaffold material (Li et al., 2023; Shu et al., 2024). The dipping/leaching method and the coagulation method are similar in principle, which is to dip and dry the mold repeatedly in the raw material solution or carry out coagulation bath to form the basic molding. Both methods could control the internal diameter, wall thickness, density, pore size, porosity and other size related parameters of the scaffold in some extent and have relatively simple operating steps, but they are difficult to accurately obtain the two-dimensional and 3D micro/nano structures, so they need to be combined with other methods to complete the micro/nano scale processing. Layer by layer assembly method is to form integral 3D tubular structures through multi-layer superposition of basic materials. Its advantages are simple preparation and flexible design of adhesive cells and molecules in each layer, but the junction between layers may be not tight and prone to cracks, affecting the performance of the scaffold (Wang et al., 2016). 3D printing (also known as additive manufacturing) is a new technology that displays the 3D shape through tomography and computer simulation, and then reconstructs the shape by bioink materials (Chen et al., 2024). However, the harsh conditions of SDVGs make the material requirements of 3D printing for this application very high, and the high costs for biological 3D printing equipments to control the micro/nano structure limit the application of this technology in SDVGs as well.

**FIGURE 1:**
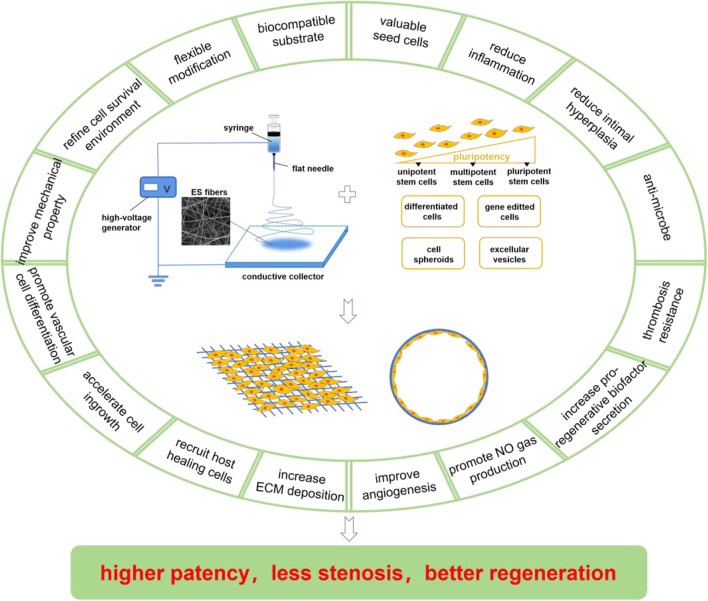
Compositions and merits of ES/SC SDVGs. The ES device is normally composed of a high-voltage generator, a syringe, a flat needle, and a conductive collector, which produces fibrous scaffolds facilely. The SCs applied in ES SDVGs contain pluripotent SCs, multipotent SCs, and unipotent SCs (with pluripotency in a descending order) and some preparations derived from them including the differentiated cells, the gene-editted cells, the cell spheroids, the extracellular vesicles, etc. The ES scaffolds provide biocampatible substrates for the SDVGs, which could realize flexible modification, refine cell survival environment, improve the mechanical property of the graft, promote vascular differention of the SCs, accelarate cell ingrowth, recruit host healing cells, increase ECM deposition, etc. The SCs provide valuable seed cells for the SDVGs, which could reduce inflammation, reduce intimal hyperplasia formation, anti-microbe, resist thrombosis formation, increase pro-regenerative biofactors and NO gas production, improve angiogenesis, etc. Some of the merits of ES scaffolds and SCs are overlapped. These merits make the ES/SC SDVGs higer patency, less stenosis and better regeneration outcome.

In contrast, ES method can avoid most of the problems of the above methods, since there are its several specific benefits. Firstly, the ES equipment and operation is simple. A typical ES device is only composed of a high-voltage generator, a syringe, a flat needle, and a conductive collector (Figure 1). The device supplies solution through the spinneret and applies high pressure to the tip, so that the charged solution accumulates electrostatic repulsion, and ejects fine fiber flow from the tip of the flat needle. Conductive collectors (collector plates or rods) with opposite charges or grounding adsorb these continuous fibers and make them overlap one another to form a highly porous network. By modifying variables such as the distance between the pin and the collector, the applied voltage, or the solution flow rate, researchers can facilely change the overall structure of the scaffold. Secondly, ES technology is flexible and universal. It can rapidly prepare a variety of natural or synthetic polymers in large scale, and has become a common method for scaffold preparation in many laboratories and even commercial companies (Huang et al., 2022). The natrual polymers used in SDVGs contain gelatin, collagen, silk fibroin, chitosan, hyaluronic acid, etc. The synthetic ones include polycaprolactone (PCL), polyurethane (PU), poly (lactide) (PLA), polyglycolic acid (PGA), poly (glyceryl carbate) (PGS), poly (lactide-co-caprolactone) (PLCL), poly (lactic-co-glycolic acid) (PLGA), polyvinyl acetate (PVA), etc. To acquire better mechanical, biocampatible or regenerative properties, these polymers could be combined in proper ratios and/or forms. Thirdly, the ES fibers are ECM-mimicking. In the field of biomedicine, most tissues and organs in the human body are similar to nano-/micro-fibers in microstructure. The ES fibers can simulate the natural structure and biological function of ECM and can be easily modified by bioactive molecules, so it is possible to repair and regenerate tissues and organs using these micro-/nano-fibers. Many ES materials also have good biocompatibility and biodegradability with large specific surface area, high porosity, and other excellent characteristics. Therefore, ES technology has attracted accumulating attention of researchers in the biomedical field and has been widely used in tissue engineering and other related aspects.

### 1.3 Stem cells loaded ES SDVGs: unique advantages

According to whether cells are introduced during preparation, SDVGs are divided into traditional tissue-engineered ones with cell modification and “*in-vivo* tissue-engineered” ones without cell modification (that is, the integration and regeneration of SDVGs are completed by using the natural physiological reaction within the organism). Due to the loaded cells with physiological activities, the cell-modifying SDVGs are expected to maintain or even enhance their anticoagulation and regeneration performance over time (La and Tranquillo, 2018; Fayon et al., 2021). Since the first cell cell-modifying SDVG was established by Weinberg and Bell in 1986, such VGs had experienced intensively improvement (Fayon et al., 2021). The focus of these VGs is rapid formation of confluent endothelial layer because it is recognised the crucial impact of this layer on thrombosis resistance. The mechanism involves that the endothelial layer is able to secret anti-coagulation sustances such as nitric oxide (NO), prostacyclin, plasminogen activator, glycosaminoglycans, etc. It is known that there are three elements for cell-modifying VGs: the scaffolds, the seed cells, and the signal molecules. The scaffolds provide the mechanical support for confining the blood flow and the backbone for cell attachment. The signal molecules, which mostly incorporated into or modified on the surface of the scaffolds, provide pro-regenerative cues for the VGs. The role of the seed cells loaded on the signal molecules-containing scaffolds is to participate into the structure of the VGs or to attract other regenerative cells into the VGs. Therefore, the selection of proper scaffolds, seed cells and signal molecules is determinant for cell-modifying SDVG design.

As previously mentioned, ES fibrous scaffolds are suitable for SDVGs. They can also be easily further empowered by adding pro-regenerative signal molecules. On the other hand, selecting suitable seed cells is another key step in SDVG construction. There is a wide range of sources for seed cells. Compared with mature somatic cells, stem cells (SCs) featuring the potential of self-renewal and directional differentiation tend to have better regeneration potential. These characteristics provide a good basis for SCs as seed cells for SDVGs. In recent years, SDVGs constructed by ES matrices plus SCs have been reported to have good regeneration outcome, showing its potential as a new generation of SDVGs. These SDVGs typically blend merits of both ES matrices and SCs, which are generally recognized as having unique advantages. This review will summarize the research progress on SDVGs that blend ES matrices and different kinds of SCs (in line with the differentiation potential of SCs from high to low in turn: pluripotent stem cells, multipotent stem cells, and unipotent stem cells) together with SC-derived preparations (such as the differentiated cells, the gene-editted cells, the cell spheroids, and the extracellular vesicles) (Figure 1), and analyze the challenges and prospect the future directions of electrospining matrix/stem cell blending SDVGs (ES/SC SDVGs).

## 2 Pluripotent stem cells in ES/SC SDVGs

Pluripotent stem cells (PSCs) are a kind of cell holding the ability to proliferate indefinitely and differentiate into all types of in-body cells. According to the sources, PSCs include embryonic stem cells (ESCs) and induced pluripotent stem cells (iPSCs). ESCs are isolated from early embryos or primordial gonads, while iPSCs are established by reprogramming somatic cells by importing specific exogenous genes or treating with a delicate set of chemical molecules, rendering them have similar properties to ESCs. PSCs are valuable cell sources for vascular and other tissue engineering for they are really abundant in sources and plastic to differentiate into multiple target cells, and what’s more for the iPSCs, they also effectively avoids immune rejection and ethical issues belonging to ESCs when implanted into homogenic hosts. It has been reported that ESCs and iPSCs can be effectively induced to differentiate into vascular endothelial cells (ECs) and smooth muscle cells (SMCs) in a suitable environment both *in vivo* and *in vitro* (Yamamoto et al., 2005; Ji et al., 2023). A number of reports have demonstrated the feasibility of PSCs in SDVGs (Ji et al., 2023). It noted that to avoid the tumorigenicity and differentiatial uncertainty, PSCs are mostly not directly implanted into the VGs, but instead its further differentiated cells.

**FIGURE 2:**
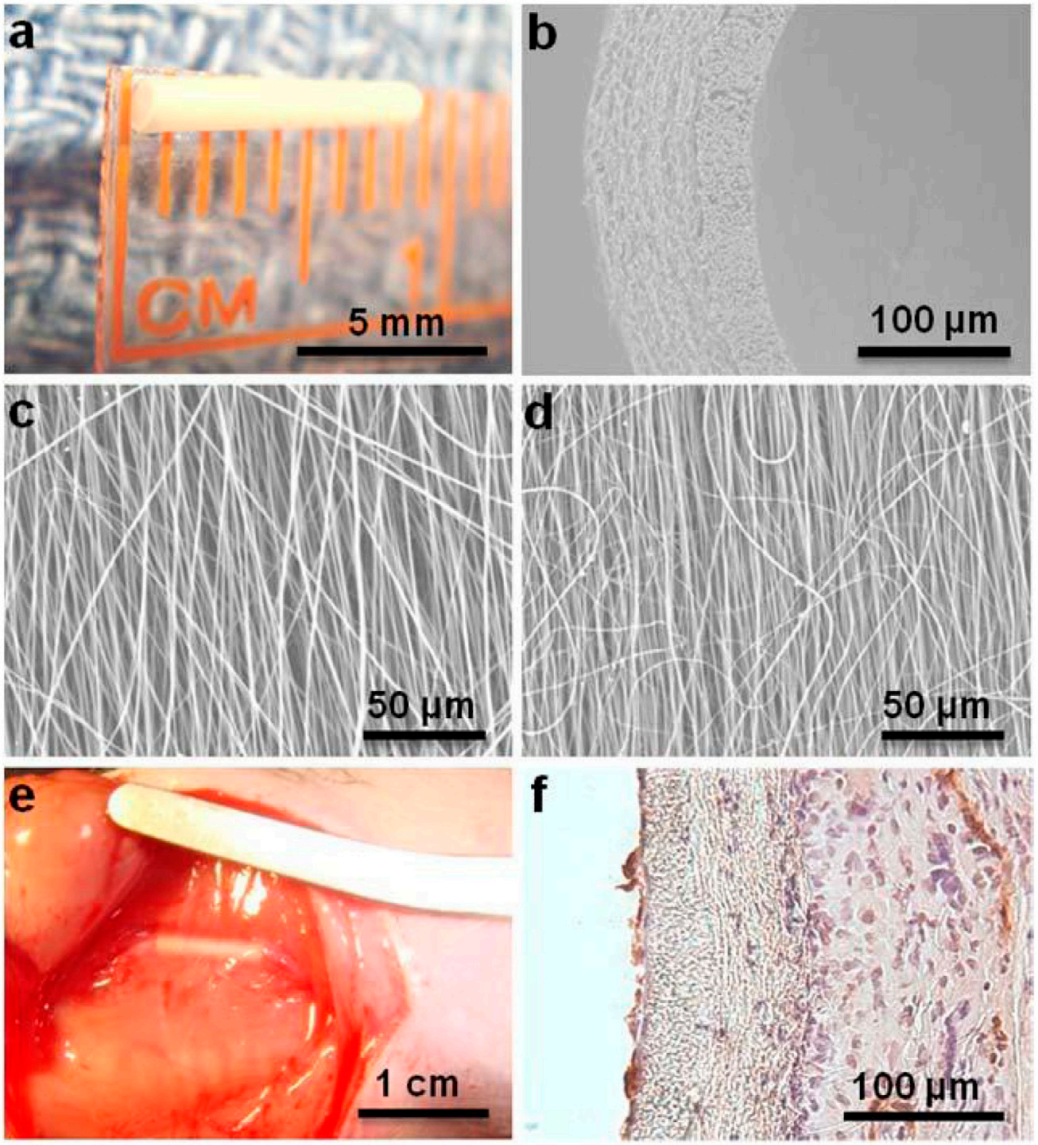
Application of pluripotent stem cells (iNCSCs herein) in ES/SC SDVGs. **(A)** ES VG with an inner diameter of 1 mm and wall thickness of 100 µm. **(B)** SEM image of a cross section of nanofibrous vascular scaffold. **(C–D)** SEM images of longitudinally aligned fibers in luminal layer **(C)** and circumferentially aligned fibers in outer layer **(D)**. **(E)** The iNCSC seeded VG after 3 months of implantation. **(F)** H&E and CD31 staining of a cross section of the VG after 3 months of implantation. Reprinted with permission from reference (Li et al., 2020). Copyright 2020, Mary Ann Liebert.

First of all, the performance of ES SDVGs may be improved by PSC-derived cells. Tang et al. constructed ES PCL randomly distributed fiber meshes with different mass denstiy (37.7 ± 16.3, 103.8 ± 16.3, 198.2 ± 40.0, and 471.8 ± 32.7 μg/cm^2^, respectively) and found the second mesh was most conductive to a variety of behaviors of human iPSC-derived MSCs (iMSCs) seeded on it, including cell adhesion, spreading, migration, contraction, proliferation, collagen secretion, actin polymerazation, as well as Yes-associated protein (YAP) nuclear import and the following transcriptional regulation. These results illustrated the link between the cell behavior and the ES mesh density, laying a foundation for vascular regeneration applications (Tang et al., 2020). Wu et al. co-cultured human umbilic vein endothelial cells (HUVECs) with iMSCs on PCL ES meshes with aligned or randomly distributed fibers. Micro vessels formed well on both meshes and the vessels on the former were also distributed along the fibers in an aligned manner. These properties provided multiple choices for the construction of pre-vascularized SDVGs (Wu et al., 2017). The graft exhibited great cell viability and compatibility, suggesting a potentially available VG for application. Li et al. applied a novel seed cell source, iPSC-derived neural crest stem cells (NCSCs) (iNCSCs), on the lumen of the ES poly (lactide) (PLA) tubular scaffolds with different stiffnesses to construct the SDVG (Figure 2) (Li et al., 2020) Compared with the soft matrix, the hard matrix drove iNCSCs to differentiate into SMCs, and TGF-β1 addition further enhanced this differentiation. The VGs were then implanted into the left common carotid artery replacement models of athymic rats for 3 months. The results showed that the ES fibers offered an excellent VG scaffold. and iNCSCs could be used as a promising autologous cell source for SDVGs when combined with physical (scaffold stiffness) and chemical (TGF-β1) signals that enhance SMC differentiation and graft mechanical strength.

Second, ES matrices provide physical cues for promoting PSCs and PSC-derived cells to differentiate into vascular cells. It was demonstrated that compared with culture dish surfaces, PCL ES mats promoted iPSC towards vascular EC differentiation (Kim et al., 2017). During a 5-day differentiation process, vascular EC markersnif were sigicantly upregulated in the ES mat culture group, including the gene expression of CD31 (∼11 folds), CD144 (>7 folds), endothelial nitric oxide synthase (eNOS) (>7 folds), protein expression of CD31 (>3 folds), nitric oxide (NO) gas production (∼4 folds). Wang et al. presented that amino acid substituted poly (organophosphazene) polymer ES matrix boosted iMSCs for mature contractile vascular SMC differentiation (Wang M. et al., 2022). The transcription level of early to late smooth muscle marker proteins on the ES fiber mats was significantly increased.

Last but not least, ES matrices provide a protective engraftment environment for PSCs or PSC-derived vascular cells. Hoveizi et al. found that compared with casted flat mats, ES PCL mats were beneficial to the adhesion and proliferation of human iPSCs (Hoveizi et al., 2015). Yu et al. prepared ES polyurethane (PU)/fibroin tubular scaffolds with parallel mechanical properties to native blood vessels, and then loaded iPSC-derived ECs (iECs) to construct SDVGs (Yu et al., 2016). Tan et al. have reported that prolonged survival time of iECs *in vivo* could be achieved by culturing them on PCL/gelatin (70:30) ES scaffolds in a wound healing model (Tan et al., 2018). Compared with the cells directly injected, the survival period of iECs implanted on the scaffold could be improved from several hours to up to 3 days, and micro blood vessel perfusion, expression of vascular endothelial growth factor (VEGF) and placental growth factor, and macrophage recruitment were increased in the healing would.

## 3 Multipotent stem cells in ES/SC SDVGs

Multipotent SCs, with a lower differentiation potential compared to PSCs, can only be specialized into one type or two closely related types of cells. Multipotent SCs are mainly isolated from different human tissues, so they are also called tissue-specific SCs. According to their tissue sources, the multipotent SCs used in ES/SC SDVGs mainly include bone marrow mesenchymal stem cells (BMMSCs), adipose mesenchymal stem cells (ADMSCs), placenta-derived mesenchymal stem cells (such as umbilical cord mesenchymal stem cells (UCMSCs) and amniotic mesenchymal stem cells (AMMSCs)), and some others.

### 3.1 Bone marrow mesenchymal stem cells

BMMSCs, one of the most intensively applied MSCs, are isolated from bone marrow tissues. As an important cell source for tissue repair, BMMSCs have been proved to have the potential to differentiate into vascular ECs and SMCs (Cho et al., 2005). Mounting reports have presented that BMMSCs have shown excellent regenerative potentials in ES/SC SDVGs.

**FIGURE 3:**
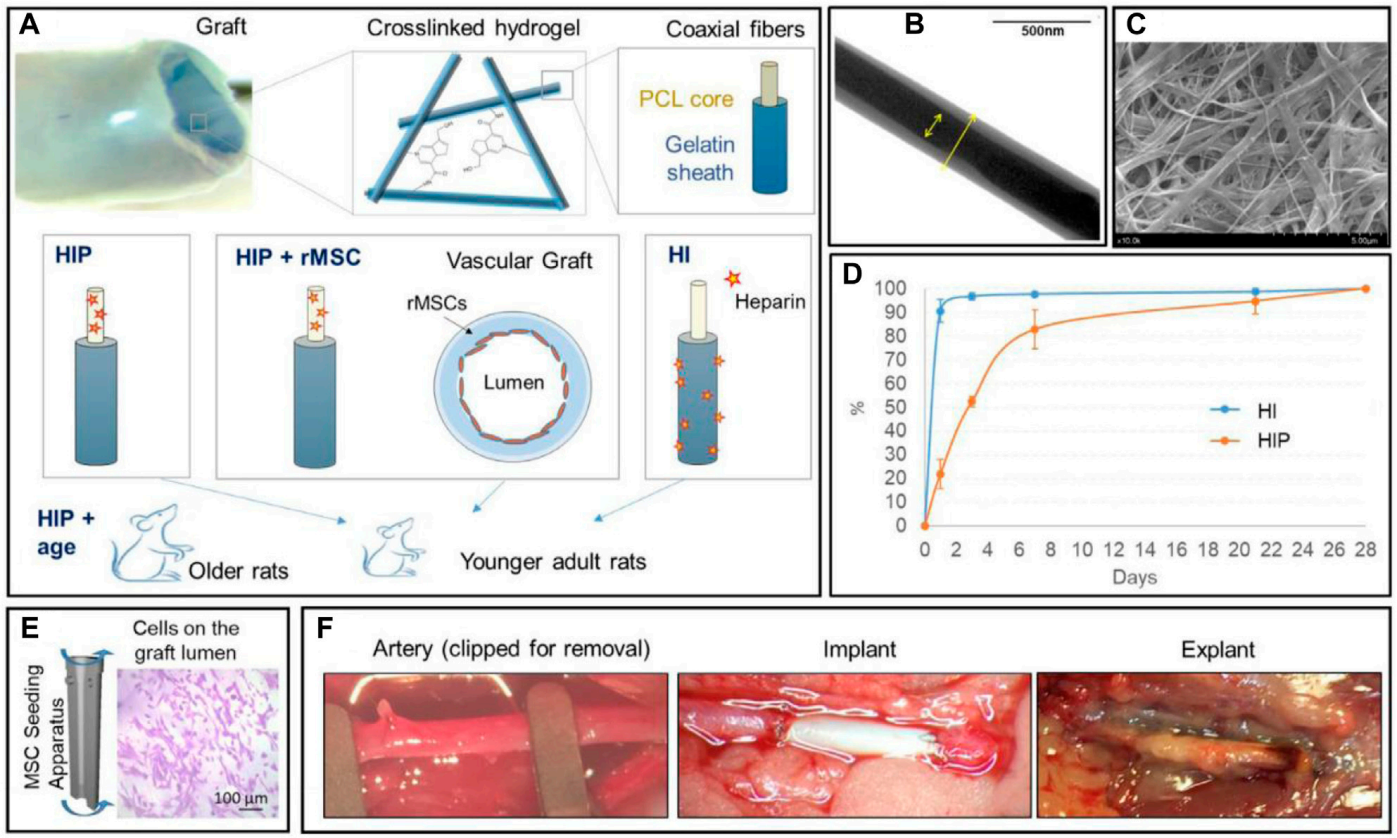
Application of multipotent stem cells (BMMSCs herein) in ES/SC SDVGs. **(A)** Illustration of arterial graft design (the top panel) and experimental group design (the bottom panel). **(B)** TEM image showing the coaxial nanostructure of PCL/gelatin fibers. **(C)** Representative SEM image of the crosslinked coaxial fiber scaffolds. **(D)** The cumulative heparin release profiles of heparin-immersed (HI) and heparin-impregnated (HIP) grafts. **(E)** Illustration of the centrifugal seeding of BMMSCs onto the graft lumen, which are visible with crystal violet stain. **(F)** Representative pictures showing the implantation and explanation of interpositional artery grafts in a rat abdominal aorta replacement model. Reprinted with permission from reference (Johnson et al., 2021). Copyright 2021, Elsevier.

In the first place, BMMSCs have shown superior performance in *in-vivo* implantation of ES/SC SDVGs. It has been reported that compared with acellular grafts, human BMMSC-seeded PLA ES SDVGs with aligned nanofibers inhibited platelet adhesion and thrombus formation, accelerated cell in-growth and extracellular matrix remodeling, and promoted organized formation of EC and SMC layers, thus assuring long-term patency of the graft when applied in an athymic rat common carotid artery replacement model (Hashi et al., 2007). Surface heparan sulfate proteoglycans of BMMSCs were evidenced to play important roles on the platelet resistance and antithrombogenic property of the graft. BMMSC (from allogeneic rat) seeding also promoted the remodeling of ES PCL/gelatin VG coated with a thin strengthening sheath of ES PCL (Figure 3) (Johnson et al., 2021) After 4.5 months of implantation in abdominal aorta replacement models in rats, compared with acellular grafts, the cell-seeded grafts showed prominent cell ingrowth and ECM deposition, in which collagen and especially elastin content and arrangement are much more similar to native arteries. It was supposed that nutritional capacity of BMMSCs to stimulate the cells of recipient rats may be the reason on the refined regeneration. BMMSCs even exhibited their effectiveness in aged animal models. Madhavan et al. implanted a multilayer, compliant SDVGs with ES fibers composed of collagen/chitosan shell encapsulating heparin core into a senescent sheep carotid artery replacement model (Madhavan et al., 2018). After 1 month, all grafts retained patents and no stenosis happened. In contrast to acellular ones, the cellular grafts showed less anastomotic stenosis and IH, as well as more EC regeneration. The results highlighted the strong healing capacity of BMMSCs. In the last example, BMMSCs were seeded on SDVGs made of a poly (urethane) urea tube by thermally induced phase separation and an external ES layer by the same material to enhance the mechanical property (Colunga and Dalton, 2018). After implanted into the abdominal aorta of rats for 8 weeks, the cellular grafts possessed continuous EC layers in the lumen and no evidence of IH, while the acellular control group showed platelet and fibrin deposition.

Dynamic perfusion/rotating bioreactors are important biomechanical mimicking devices in blood vessel study and BMMSCs have also shown pro-regenerative effects on the ES/SC SDVGs cultured in these devices. For instance, Rekabgardan et al. prepared human BMMSC-seeded PGS/PU ES scaffolds with an ideal elastic modulus close to that of human saphenous vein and with anti-platelet adhesion property (Rekabgardan et al., 2022a). When applying shear stress of ∼15 dyn/cm for 24 h to the scaffold in a perfusion bioreactor, the expression of VEGFR-2, vWF and PCAM-1 in BMMSCs was significantly increased compared with that in static culture. Therefore, the scaffold could support the endothelial differentiation of the MSCs under shear stress without any growth factors. In another study, Kim et al. placed the human BMMSC-seeded PLCL ES SDVGs in a perfusion system to test the effect of two different force giving modes (SS only mode or SS + CS mode) on cell differentiation (Kim et al., 2016). They found that in the latter mode, EC markers expressed more lastingly compared to the former, indicating that SS + CS was a better condition for vascular EC differentiation on the scaffold. In the third study, Wang et al. setup PECUU ES SDVGs seeded with BMMSCs by electrospray on a rotating bioreactor (Wang et al., 2013). After 2 weeks of culture, cells maintained their viability and proliferation. These properties, together with the good antithromboticity, fast fabrication within 40 min, and similar mechanical properties to natural arteries, indicated this design might be promising in the regeneration of functional vessels.

The regenerative effect of BMMSCs on ES matrices is supported by membrane culture experiments as well. Firstly, ES mats are supportive substrates for BMMSC growth. It has been reported that ES fibroin nanofibers supported human BMMSC attachment, spreading and growth (Jin et al., 2004). In another case, eNOS gene modified rat BMMSCs could also maintain good activity when they were seeded on ES poly (propylene carbonate) nanofibrous mats (Zhang et al., 2006). The mats promoted the survival and proliferation of transfected MSCs, and the level of NO produced by MSCs was rather high, similar to that of freshly collected arterial ECs. Adequate modification of ES materials would improve their performance. It has been illustrated that proliferation of BMMSCs and HUVECs in egg white/polyvinyl acetate (PVA) ES mats was promoted by the addition of graphene oxide (GO), suggesting biocompatibility of the mats was enhanced by GO modification (Wang et al., 2021). Similarly, a SDVG ES PLCL modified by substance P (SP) maintained biological activity and homing of human BMMSCs (Shafiq et al., 2015). In addition, RGD (Arg-Gly-Asp) peptides fixed on the PCL/gelatin ES nanofibers improved the cell adhesion and proliferation (Mota et al., 2014). Intriguingly, regenerative effects of BMMSCs on ES mats were also influenced by ES fiber diameters. Human BMMSCs were planted on PLA ES mat with different diameters (440, 865 and 2,142 nm), and compared with control group PLA casting flat mat and cultured on the surface of polystyrene, the cells showed ECM secretion in hBMSCs grown on PLA ES mat were higher than those on PLA mats (Liu et al., 2022). The levels of soluble collagen and sulfate mucopolysaccharide decreased with the increase of PLA fiber diameter. Therefore, nanoscale ES PLA scaffolds seemed to be more suitable for promoting the formation of ECM components. ES matrices are in support of endothelial differentiation of BMMSCs. Rekabgardan et al. cultured BMMSCs on a double-layered mat design with good mechanical properties, hydrophilicity and biodegradability, whose upper layer was ES PGS/PU and lower layer was ES pure PU (Rekabgardan et al., 2022b). The expression of platelet endothelial cell adhesion molecule-1, von Willebr factor (vWF) and VEGF receptor two showed that the ES scaffold supported the endothelial differentiation of the seeded BMMSCs. Others found that the proliferation of BMMSCs and endothelial differentiation on PLA/collagen nanofibers was higher than those on pure PLA nanofibers (Jia et al., 2013). Thus, collagen co-ES may further improve the performance of polymer ES SDVGs. Likewise, ES mats could be used for directing SMC differentiation of BMMSCs. ES mats composed of fibers containing PCL/gelatin as the shell and silk fibroin/VEGF as the core, human BMMSCs cultured on the mats showed the differentiation of SMCs through the controlled release of VEGF in core/shell nanofibers (Ezhilarasu et al., 2019). Lastly, ES mat stiffness affected the BMMSC differentiation. Pectin hydrogel nanofiber mats with different oxidation degrees (25% and 50%) were prepared by periodate oxidation, ES and dihydrazide adipate crosslinking (Li et al., 2019). The stiffness of the mats was controlled by adjusting the oxidation and crosslinking levels of pectin gel. Although human BMMSCs cultured on both pectin gel fiber mats for 14 days had high viability, the harder scaffold promoted the differentiation of MSCs into vascular SMCs, whereas the softer fibrous mat promoted the differentiation of MSCs into ECs.

### 3.2 Adipose mesenchymal stem cell

ADMSCs, another widely used MSCs, are isolated from adipose tissues. Since they can be acquired from the discarded adipose of liposuction, ADMSCs have the advantages of rich sources, easy access, and low invasion. ADMSCs, able to be differentiated into vascular ECs and SMCs under proper conditions, (Yogi et al., 2021), have been proved an excellent cell source in ES/SC SDVGs.

ES matrix provided a sound support for growth and vascular lineage differentiation of ADMSCs. For instance, lysozyme-conjugated heparin functionalized PLA and segmented PU (50:50) ES scaffolds increased hydrophilicity and water absorption of culture surface, and promoted the adhesion and proliferation of human ADMSCs. In addition, the functionalization endowed the scaffold great anti-coagulation, anti-microbe, and hemocampatibility (Caracciolo et al., 2017). Sun et al. coated rat ADMSC-derived EPCs and PCL ES matrix with sodium alginate solution and then the EPCs were immediately cross-linked to the ES matrix to form a confluent layer. Compared with the EPCs and ES matrix without sodium alginate coating (namely, natural sedimentation), the coated group exhibited much quicker adhesion and proliferation, suggesting that alginate modification might accelerate the formation of endothelial layer of SDVGs (Sun et al., 2015). Such a usage of alginate would also circumvent its known drawback that alginate is not conducive for cell attachment (Raus et al., 2021). In a following work, it was presented that ADMSC proliferation could be realized on polyhydroxybutyrate (PHB)/poly (3-hydroxybutyrate-co-3-hydroxyvalerate) (PHB-HV) (30:70) ES matrix for up to 21 days. Moreover, endothelial differentiation of ADMSCs was achieved on the matrix (Zonari et al., 2012). Smooth muscle differentiation on ES matrix were also been completed. It was found that mechanical stretching and biochemical factors played a synergistic role on the smooth muscle differentiation of human ADMSCs in an elastic PLCL (50:50) ES scaffold (Park et al., 2012). In the culture without stretching, the smooth muscle differentiation factors had a significant elevation on α-smooth muscle actin (α-SMA) expression, but myosin heavy chain (MHC) expression were not affected, whereas in the stretching culture, the expression of α-SMA and MHC was both highly lifted by smooth muscle differentiation factors. This study provided a useful method for the application of SCs in mechanically activated tissue engineering.

On the other hand, the function of vascular ECs can be enhanced when co-culturing with ADMSCs on ES matrices. For instance, co-culture of ADMSCs and HUVECs on ES PCL/gelatin scaffolds could significantly promote the proliferation and angiogenesis of HUVECs. HUVECs co cultured produced mature and functional microvessels, as well as higher expression of vascular markers such as platelet endothelial cell adhesion molecule-1 and calyx protein (Kook et al., 2018). Both the direct interaction with ADMSCs and the paracrine signals from ADMSCs are the key factors in microvessel assembly. By producing the same material-derived ES scaffolds plus heparin modification, researchers illustrated that the 7-day co-culture enhanced CD31 expression of HUVECs and healthy cobblestone HUVEC morphology could be achieved, indicating that the co culture system could promote the endothelialization of vascular matrix (Joshi et al., 2020). Material composition also played roles on the co-culture system. Kenar et al. prepared a kind of PLCL/collagen/HA (90/9.5/0.5 w/w/w) ES scaffold. Compared with the bare PLCL ES scaffold, the collagen and HA addition increased the total length of vascular network in the co-culture (1.6 folds), improved the water absorption capacity (from 66% to 103%), and lowered Young’s modulus (from 1.31 to 0.89 MPa) (Kenar et al., 2019). It was noteworthy that the collagen and HA were derived from xenogeneic human umbilical cord, which could avoid the immune reaction and the spread of animal borne diseases of animal-derived ones. Overall, the co-culture system of HUVECs and ADMSCs might have the advantages of rapid endothelialization and promoting angiogenesis.

Interestingly, ES matrix also benefit for the functioning of the ADMSC-derived spheroids, another novel important application form of ADMSCs, which are famous for their potent angiogenic factor release (Lee et al., 2020). To achieve the sustained release of these factors, Lee et al. seeded the spheroids on a 3D printed alginate mat with micro-sized grooves and ridges and then employed ES alginate nanofibers to firmly entrapped the spheroids. Compared to the single-type cell-seeded counterpart, co-culture of HUVECs formed much more capillary-like structures in the spheroid-seed 3D printing/ES alginate scaffold. These results suggested a new system for SC-containing ES VGs.

### 3.3 Placenta-derived mesenchymal stem cells

Placenta-derived MSCs such as UCMSCs and AMMSCs, are MSCs isolated from placenta, a traditionally clinical waste tissue abandoned by pregnant women after delivery. Thus, they have many advantages like high activity, low immunogenicity, source abundance, easy collection and transportation, and no ethical disputes. It has been proved that UCMSCs and AMMSCs have the potential to differentiate into vascular ECs (Chen et al., 2009).

A few studies have demonstrated the positive regenerative potential of these MSCs in ES/SC SDVGs. For instance, chitosan (CS)/PVA ES nanofiber scaffolds were prepared and confirmed good crystalline properties, hydrophilic properties, biodegradability, swelling and mechanical properties (Agrawal and Pramanik, 2016). UCMSCs implantation tests demonstrated that the scaffold has sufficient biocompatibility and cell supporting properties *in vitro*. As for AMMSCs, ES PLCL scaffolds was used as the substrate to compare with the commercially available collagen/elastin scaffold (matriderm) (Vonbrunn et al., 2020). Human AMMSCs adhered on and infiltrated well into both scaffolds and on the third day, more cells were observed on PLCL than on matriderm. The cell seeded patches were used to cover the skin wound in mouse models. PLCL scaffolds showed higher cell retention than matriderm. Therefore, SDVGs based on human AMMSCs/ES complex had great potential. In the end, it is worth mentioning that MSC-derived extracellular vesicles (EVs) have been incorporated into ES SDVGs. EVs are nanoscale vesicles containing bioactive molecules such as proteins and nucleic acids, which can recapitulate the major functions of origin cells. EVs of human placenta-derived MSCs (the specific portion of placenta was not described) were used to decorate heparin-modified ES PCL SDVGs (Wei et al., 2019). The graft showed significant enhancement of vascular cell regeneration, inhibition of inflammation, thrombosis and calcification, and hence great patency in a 3-month rat abdominal artery replacement model with high cholesterol. Bioactive factors in the EVs like miR-145, miR-126, and VEGF played a crucial role in vascular regeneration.

### 3.4 Other multipotent stem cells

There are some other kinds of multipotent stem cells applied in ES/SC SDVGs, including pericytes, dental pulp MSCs (DPMSCs), embryonic multipotent mesenchymal progenitor cells (EMMPCs), and muscle-derived stem cells (MDSCs). Besides, we also included application of bone marraw mononuclear cells (BMMNCs) in ES/SC SDVGs in this review, because BMMNCs show similar functions as MSCs in SDVG settings and contain MSC fractions.

Pericytes are cells that surround vascular ECs in capillaries and veins, which usually perform a variety of functions in angiogenesis, including vascular stability, matrix protein synthesis, tissue homeostasis, and macrophage-like properties. They were considered to be a MSC recently, due to their potential to differentiation into a variety of cell types, including vascular ECs and SMCs (Krawiec and Vorp, 2012). He et al. deposited ES poly (ester carbamate) urea (PEUU) nanofibers on the thermally induced phase separation tubular scaffold and pericytes were seeded on the scaffolds and cultured in a rotating flask for 2 days before being implanted into rat aortic replacement models (He et al., 2010). 8 weeks post-implantation, compared with SDVGs without cells, the cellular grafts showed a much higher patency rate (100% vs. 38%), and extensive EC/SMC layer remodeling and collagen/elastin deposition, as well as evenly distributed pericytes sustaining their intrinsic phenotype markers.

Dental pulp MSCs (DPMSCs) are obtained from soft teeth pulp tissue within the dental pulp cavity. Teeth that naturally fall off in children and wisdom teeth that need to be extracted in adults both contain abundant DPMSCs. Braghirolli et al. found that copolymers with different trimethylene carbonate/lactide ratios ES SDVGs supported the adhesion and growth of DPMSCs, umbilical cord blood EPCs, and primary aortic SMCs, and promoted the differentiation of DPMSCs into vascular SMCs (Braghirolli et al., 2019). The same group also evaluated the effect of DPMSCs on another kind of SDVGs composed of heparin/VEGF-modified PCL ES fibers. Studies have shown that heparin and VEGF functionalization exhibited antithrombotic properties and the ability to promote DPMSCs growth and umbilical cord blood EPCs endothelialization, which were conducive to the formation of endothelial cell layer of vascular scaffolds and the regeneration of damaged vessels.

Embryonic multipotent mesenchymal progenitor cells (EMMPCs) are extracted from early embryo, and have very high proliferation and differentiation activity. Kiros et al. prepared tubular ES polyester amide fibers from biodegradable biomaterials derived from L-phenylalanine, and seeded with mouse EMMPCs and ES fiber scaffold (Kiros et al., 2020). The scaffold showed the ability to differentiate EMMPCs into vascular SMCs, and the differentiated MSCs produced crucial extracellular proteins such as elastin and fibrillin-1.

Muscle-derived stem cells (MDSCs) are a kind of adult stem cell found in skeletal muscle, serving as important coordinators for muscle regeneration and repair. Nieponice et al. constructed PEUU tubes by thermally induced phase separation (TIPS) with ES reinforcing layers outside, and seeded with MDSCs (Nieponice et al., 2010). The construct was implanted into the abdominal aorta of rats for 8 weeks. The cell inoculated constructs showed a higher patency rate than the non-seeded control: 65% (cellular ES + TIPS group) vs. 53% (cellular TIPS group) vs. 10% (acellular TIPS group). The mechanical failure rate of tips stent was 50%, aneurysms were formed, but no expansion was observed in the mixed stent. SMC and EC layers within the cellular SDVG, while the acellular control showed platelet and fibrin deposition.

At last, BMMNCs are cells with a single nucleus in bone marrow, which are a mixed cell populations typically including monocytes and lymphocytes. They have strong cellular immunologic functions and have been employed in the application of SDVGs. It seems that the function of BMMNCs in SDVGs resembles BMMSCs. In PLCL ES SDVGs, the seeded BMMNCs could prevent stenosis by regulating the function of host macrophages and platelets, indicating that BMMNC inoculation into the ES scaffold potentiated a favorable SDVGs (Yao et al., 2018). Fukunishi et al. fabricated mouse BMMNC-seeding ES polyglycolic acid (PGA)/PLCL SDVGs and transplanted them into mouse infrarenal inferior vena cava replacement models for 6 months (Fukunishi et al., 2017). Compared to acellular alternatives, the cell-seeding ones greatly improved the patency (9/10 vs. 1/10) and showed increased EC/SMC coverage and collagen-rich ECM deposition. The mechanism was linked to the fact that the BMMNCs could decrease the activation of platelets and increase the M2 macrophage content.

## 4 Unipotent stem cells in ES/SC SDVGs

Unipotent stem cells, possessing even lower differentiation potential compared to multipotent stem cells, can only be specialized into one defined type of mature cells. Unipotent stem cells used in SDVGs mainly refer to endothelial progenitor cells (EPCs) and smooth muscle progenitor cells (SMPCs) that can exclusively differentiate into mature vascular ECs and SMCs, respectively. These progenitor cells mainly originate from bone marrow and exist in a small amount in the peripheral blood in adults (Colunga and Dalton, 2018). Under the stimulation of physiological or pathological factors, they can be mobilized from bone marrow to peripheral blood to participate in the repair of injured blood vessels. In ES/SC SDVG applications, there have been plentiful studies regarding EPCs due to the close relationship between EPCs and ECs, while the applications on SMPCs are still underexplored. It is of special note that as cells existing in the blood circulation, EPCs or SMPCs could also be recruited to the SDVGs *in situ* after graft transplantation.

EPCs have played pro-regenerative roles in ES/SC SDVGs under static culture. It is presented that EPCs can adhere and grow on the PHA (PHB and PHBV) ES mats without cytotoxicity and normal marker expression and biological function during a 7-day culture (Yao et al., 2018). The PHA ES mats also had good mechanical properties. Therefore, they might be ideal biopolymers for EPC-seeded SDVGs. In another study, ES polymer scaffolds were coated with decorin, an important leucine rich small proteoglycan, widely existing in the ECM of many organs and tissues (Hinderer et al., 2018). Decorin-coated scaffold promoted the proliferation of EPCs seeded, compared to uncoated control and to scaffold coated by SDF-1α, another frequently used coating agent for attracting EPCs. The researchers also found that decorin reduced T cell responses and attracted innate immune cells, which might be beneficial for ECM remodeling. In a third study, PLCL ES scaffolds modified with gelatin could not only maintain the adhesion and proliferation of EPCs, but promote their endothelial differentiation (Zhang et al., 2018). Dual component coating seemed to be a novel strategy to improve the performance of EPC-loaded ES scaffolds. Braghirolli et al. reported that PCL ES fiber scaffolds were functionalized with heparin and VEGF, and human EPCs were seeded on the scaffolds to form an endothelial layer (Braghirolli et al., 2017). The scaffolds showed mechanical properties compatible with natural arteries and the proliferation of EPCs was promoted by combination of heparin and VEGF coating. Chen et al. encapsulated heparin and VEGF into the core of poly PLCL ES nanofibers to test their feasibility in SDVGs. The released heparin performed well as an anticoagulant, and the released VEGF promoted the growth of EPC on the fiber scaffold, indicating that the scaffolds have great potential in the development of SDVGs (Chen et al., 2015). In the fourth case, zwitterionic poly (carboxybetaine methacrylate) (PCBMA) and TPS peptide dual modified ES PCL mats were prepared (Li et al., 2013). PCBMA could inhibit platelet adhesion and improve the hydrophilicity, when TPS peptide not only maintained platelet adhesion resistance, but showed specificity in EPC capture. Thus, the ES scaffolds had the potential to be used as a vascular graft for rapid endothelialization. At the end, Dai et al. have encapsulated polyethylene glycol (PEG) coated ceria nanoparticles and VEGF into PU ES scaffolds (Dai et al., 2017). The sustainably released the nanoparticles retained the anti-apoptotic effect induced by oxidative stress to EPCs and enhanced the effect of VEGF on the mobilization and differentiation of EPCs. This scaffold had the potential to achieve *in situ* endothelialization.

EPCs seeded ES/SC VGs have also demonstrated their application potential in the perfusion culture system. For instance, lecithin modified PCL ES SDVGs has good biocompatibility and in a perfusion bioreactor, the seeded peripheral blood derived EPCs had the ability to differentiate into mature vascular ECs under the effect of mechanical factors and VEGF (Xing, 2008).

Furthermore, EPC-derived ECs are a novel seed cell source for ES SDVGs. Ju et al. developed a type of ES double-layer SDVG, and autologous EPC-derived ECs and antologous SMCs were employed to form structurally mimicking VGs (Ju et al., 2017). Following pretreated in a pulsatile bioreactor for layer fusion, the completely cellular vascular structure was tested in the sheep carotid artery replacement model, the cellular structure maintained structural integrity with a high degree of graft patency, and did not cause inflammatory reaction during 6 months in sheep. It has been demonstrated the feasibility of EPC-derived EC to produce functional endothelium on biodegradable polyester elastomer poly (1,8-octyl glycol-co-citrate) (POC) ES matrix (Allen et al., 2010). The phenotype and function of the differentiated cells showed characteristic cobblestone morphology of ECs and positive staining of EC markers such as vWF, vascular endothelial cadherin, Flk-1 and CD31. In addition, the derived ECs cultured on POC ES fiber scaffolds expressed eNOS levels similar to those of aortic vascular ECs.

**FIGURE 4:**
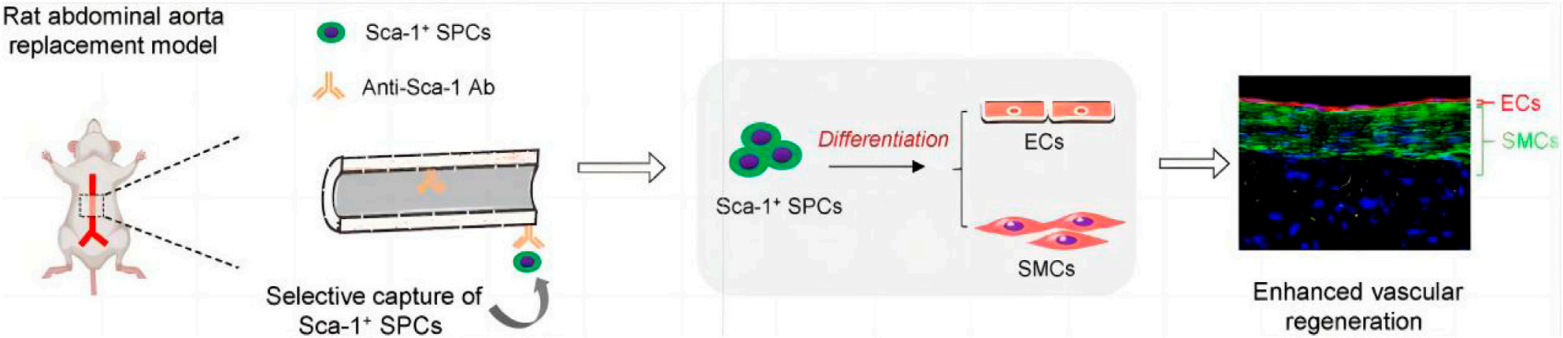
Application of unipotent stem cells (SMPCs herein) in ES/SC SDVGs. Reprinted with permission from reference (Wang H. et al., 2022). Copyright 2022, E.lsevier.

It is worth noting that the unipotent SCs would not only be pre-seeded before SDVG implantation as above mentioned, but be recruited *in situ* onto the SDVGs in hosts, because EPCs and SMPCs are circulating cells in blood. For instance, stromal cell derived factor 1α (SDF-1α) and VEGF loaded ES PU SDVGs were found to promote the adhesion and endothelial differentiation of EPCs *in vitro*. When the grafts were engrafted in the canine femoral artery for 6 months, the SDVGs showed excellent compliance, histocompatibility, and endothelialization of the attracted EPCs (Guo et al., 2017). Compared SDVGs without cytokines, those containing cytokines showed an extremely higher long-term patency rate (67.5% vs. 0%). In a second example, PLCL ES SDVG was immobilized with the cyclic RGD peptide cGRGDdvc (LXW7) peptides, an integrin α_V_β_3_ inhibitor with strong and precise EPC and EC capture function. Unlike traditional RGD peptides, LXW7 containing a circular RGD domain, showed much weaker binding with platelets, but much stronger binding affinity with EPCs and ECs, and no binding with inflammatory monocytes (Hao et al., 2020). After implanted into the left common carotid artery of rats, LXW7-modified grafts showed much less thromobosis, more new capillary formation in the vascular wall, and especially much higher patency rate (5/6 vs. 1/6) after 6 weeks, compared to unmodified ones. Wu et al. tried to recruit EPCs by conjugating protrusion protein-1 derived VEGF binding peptides and SDF-1α peptides in PCL ES SDVGs to improve the performance of the grafts (Wu et al., 2023). Compared with pure PCL transplantation, dipeptide modified transplantation showed good patency and tissue regeneration at 4 weeks after implantation, as indicated by stem cell recruitment, rapid endothelialization and formation of functional SMC layer. ES PCL grafts were functionalized by immobilizing biotin/avidin coupled stem cell antigen-1 (SCA-1) antibody, which was proved to specifically recruit SCA-1 positive SMPCs under static and dynamic flow culture (Figure 4) (Wang H. et al., 2022) In a rat abdominal aorta replacement model, the grafts initiated rapid re-endothelialization and smooth muscle regeneration by actively recruiting and capturing SMPCs from resident tissues and circulation, finally forming new tissues that were very structurally similar to natural arterial tissues. In another study, Yu et al. coated heparin on ES SDVGs and then stably grafted SDF-1α on heparin (Yu et al., 2012). SDF-1α increased the recruitment of both SMPCs and EPCs to the lumen, and SMPCs differentiated into SMCs in rat models and *in vitro* under static and flowing conditions, thus accelerating endothelialium and smooth muscle formation of the grafts. In addition, SDF-1α immobilized grafts had significantly higher elasticity after remodeling. Despite this, the limited remodeling of the electrospun layer in this study would be noted, which would be a challenge for many current studies.

## 5 Concluding remarks

SC and ES represent the cutting-edge technologies with practical medical promise in the fields of cell therapy and biomaterial, respectively. Their advancement has brought new hopes for many refractory diseases. SC-based regenerative medicine is called as the “third medical revolution” due to the self-renewal, differentiation, and secretion features of SCs. SCs have received extensive attention in the field of SDVG for they can offer excellent seed cell sources (Askari et al., 2017). ES is a simple, universal and large-scale material preparation technology developed in recent years. It can realize good control of material fibers and prepare ECM-like structures. ES substrates have been recognized as excellent materials for SDVGs (Wang et al., 2023). As summarized above, the ES/SC blending SDVGs may combine the advantages from both sides, which may contribute to the address of the core challenges in SDVG field, such as lumen stenosis, intimal hyperplasia, and occlusion, providing a new idea for the construction of SDVGs with compelling functions. In such VGs, ES scaffolds and SCs form an organic unity. The ES matrices provide biocampatible substrates for the SDVGs, which could realize flexible modification, refine cell survival environment, improve the mechanical property of the graft, promote vascular differention of the SCs, accelarate cell ingrowth, recruit host healing cells, increase ECM deposition, etc. While, the SCs provide valuable seed cells for the SDVGs, which could reduce inflammation, reduce intimal hyperplasia formation, anti-microbe, resist thrombosis formation, increase pro-regenerative biofactors and NO gas production, improve angiogenesis, etc. Some of the merits of ES scaffolds and SCs are overlapped. Collectively, these merits make the ES/SC SDVGs higer patency, less stenosis and better regeneration outcome (Figure 1).

Despite this, there are still many challenges for ES/SC SDVGs in preclinical studies and there is still no clinical trials regarding ES/SC SDVGs registered on the ClinicalTrials.gov website, suggesting the difficulty and researchers’ discretion to apply this kind of SDVG to clinical trials. The major translational challenges come from the fact that the long-term patency and regenerative outcome of this SDVG in animal models are still underexplored and the action mechanism of the SCs in such VG regeneration is largely unclear. Future prospects for overcoming these challenges are also proposed here. These challenges and prospects are described from three aspects:

i) The seed cell. Although SCs show excellent regeneration ability, they also have different degrees of shortcomings. The intrinsic problems for ESCs are the ethical problems for obtaining them by destroying early embryos and their tumorigenicity. iPSCs effectively avoids the immune rejection and ethical problems in ESCs, and can also retain a patient’s healthy or diseased phenotype for future personalized therapy development. They however, face problems including: 1) reprogramming efficiency is too low; 2) the optimal combination of inducible factors remains to be studied, and the insertion of exogenous vectors and genes has potential tumorigenicity; 3) the safety and reliability evaluation system for clinical application of iPSCs needs to be improved; 4) the mechanism of transcription factors reprogramming somatic cells into iPSCs is not yet clear. One of the effective use fashions for ESCs and iPSCs to avoid tumorigenicity is to differentiate them into down-steam cells. Simultaneously, studies on the transforming efficiency improvement for iPSCs and mechanism thereof should be strengthened. For BMMSCs and EPCs, their content is rather low in bone marrow/peripheral blood, which restricts their clinical applications, so it is necessary to isolate, purify and expand the two SCs *in vitro* to reach the required order of magnitude. In addition, invasive acquisition and too complex purification steps further increase the obstacles for BMMSC and EPC application. Besides finding better expanding and extracting methods for them, exploiting SCs without these issues is another strategy. Non-invasively available SCs such as UCMSCs, AMMSCs, and DPSCs, are excellent alternative cell sources. Another fascinating strategy to fully make use of EPCs is to capture them *in situ* and thus better recruiting agent is needed. Furthermore, to improve the performance of SCs, gene editting can be used to enhance their activity. From another angle, each SC has its unique advantages. Thus, exploiting the advantages of existing stem cells for proper application condition is also one of the solutions. For instance, the multipotency of ADMSCs has been proved to be independent of the age of the donor compared to BMMSCs and EPCs (Zhang et al., 2011; Pashneh-Tala et al., 2016). Since most vascular transplantation operations are performed in elderly patients, the use of ADMSCs may have special advantages. Finally, the use of new forms of SCs exosomes or spheroids/organoids to replace the corresponding stem cells has also shown promise.
ii) The ES system. The composition, structure and configuration of innovative materials are also conducive to the improvement of the performance of ES SDVGs. For example, the application of some ES materials will promote the transformation of macrophages from M1 type to M2 type, alleviate the inflammatory reaction of the tissue, and facilitate angiogenesis; (Wang et al., 2014); the preparation of SDVGs with spiral-like blood flow conduits will help to reduce the formation of thrombosis and IH (Li et al., 2022). The control of fiber morphology, the increase of fiber uniformity, and the reduction of environmental requirements and raw material limitations are the important directions of ES system improvement. In addition, the surface modification of ES substrates is innovated to be more conducive to the differentiation of SCs into ECs or angiogenesis. All of these may helpful to improve the long-term patency of ES/SC SDVGs.
iii) The interaction between SCs and the ES matrices. This interaction plays an important role in the fusion between SCs and ES materials, but much is still unclear for specific SC and specific ES material. It involves the processes of cell expansion, migration, and differentiation, as well as the mechanical, chemical signals and surface effects of scaffolds. It has been reported that microstructure of the ES material will affect a series of reactions such as the proliferation and differentiation of SCs (Nemani et al., 2018). Therefore, strengthening this research will help to find a better method to improve the patency of SDVGs or a new target to construct SDVGs.

Although still in its initial stages, the ES/SC SDVGs have been presented a promising candidate for next-generation SDVGs. Longer-term preclinical studies and more deeper fundamental researches are needed to compel the arrival of the clinical trials. In this course, a variety of challenges are to be overcome, especially how to choose proper combinations of SC source and ES material in conjugation with modifications to the point. We believe that with the continuous exploration of researchers, the ES/SC SDVGs will be closer and closer to the performance of natural blood vessels with high patency and tissue integrity, and provide a significant substitution for damaged small diameter blood vessels.

## Figures and Tables

**Table:** 

**CVD**	Cardiovascular disease
**VG**	vascular graft
**SDVG**	small diameter vascular graft
**3D**	three-dimensional
**IH**	intimal hyperplasia
**ES**	electrospinning
**SC**	stem cell
**ES/SC SDVG**	electrospining matrix/stem cell blending SDVG
**PCL**	polycaprolactone
**PSC**	pluripotent stem cell
**iPSC**	induced pluripotent stem cell
**ESC**	embryonic stem cell
**EC**	endothelial cell
**SMC**	smooth muscle cell
**MSC**	mesenchymal stem cell
**PU**	polyurethane
**iEC**	iPSC-derived EC
**NCSC**	neural crest stem cell
**iNCSC**	iPSC-derived NCSC
**PLA**	poly (lactide)
**PGA**	polyglycolic acid
**TGF-β1**	transforming growth factor β1
**ECM**	extracellular matrix
**eNOS**	endothelial nitric oxide synthase
**NO**	nitric oxide
**iMSC**	iPSC-derived MSC
**VEGF**	vascular endothelial growth factor
**PGS**	poly (glyceryl carbate)
**PLCL**	poly (lactide-co-caprolactone)
**PLGA**	poly (lactic-co-glycolic acid)
**PVA**	polyvinyl acetate
**GO**	graphene oxide
**SP**	substance P
**vWF**	von Willebr factor
**PHB**	polyhydroxybutyrate
**PHB-HV**	poly (3-hydroxybutyrate-co-3-hydroxyvalerate)
**α-SMA**	α-smooth muscle actin
**MHC**	myosin heavy chain
**HA**	hyaluronic acid
**CS**	chitosan
**EV**	extracellular vesicle
**DPMSC**	dental pulp MSC
**MDSC**	muscle-derived stem cell
**BMMNC**	bone marraw mononuclear cell
**EMMPC**	embryonic multipotent mesenchymal progenitor cell
**PEUU**	poly (ester carbamate) urea
**TIPS**	thermally induced phase separation
**EPC**	endothelial progenitor cell
**SMPC**	smooth muscle progenitor cell
**SDF-1α**	stromal cell derived factor 1α
**SCA-1**	stem cell antigen-1
**PCBMA**	poly (carboxybetaine methacrylate)
**PEG**	polyethylene glycol
**POC**	poly (1,8-octyl glycol-co-citrate)
**TEM**	transmission electron microscope
**SEM**	scanning electron microscope
**CD**	cluster of differentiation
**BMMS**	bone marrow mesenchymal stem cell
**ADMSC**	adipose mesenchymal stem cell
**UCMSC**	umbilical cord mesenchymal stem cell
**AMMSC**	amniotic mesenchymal stem cell
**HI**	heparin-immersed
**HIP**	heparin-impregnated
**VEGFR-2**	vascular endothelial growth factor receptor-2
**PECAM-1**	platelet endothelial cell adhesion molecule
**PECUU**	polyethercarbonate urethane
**HUVEC**	human umbilic vein endothelial cell
**RGD**	arginine-glycine-aspartic acid
**LXW7**	the cyclic RGD peptide cGRGDdvc
**TPS**	tissue polypeptide specific antigen
**Flk-1**	fetal liver kinase 1
**YAP**	Yes-associated protein
